# Disparity of Cervical Cancer Risk in Young Japanese Women: Bipolarized Status of HPV Vaccination and Cancer Screening

**DOI:** 10.3390/vaccines9030280

**Published:** 2021-03-19

**Authors:** Mariko Taniguchi, Yutaka Ueda, Asami Yagi, Ai Miyoshi, Yusuke Tanaka, Ryoko Minekawa, Masayuki Endo, Takuji Tomimatsu, Kei Hirai, Tomio Nakayama, Tadashi Kimura

**Affiliations:** 1 Department of Obstetrics and Gynecology, Graduate School of Medicine, Osaka University, 2-2, Yamadaoka, Suita 565-0871, Osaka, Japan; mariko27qoo@gyne.med.osaka-u.ac.jp (M.T.); a.yagi@gyne.med.osaka-u.ac.jp (A.Y.); aimiyoshi20090808@yahoo.co.jp (A.M.); tomimatsu@gyne.med.osaka-u.ac.jp (T.T.); tadashi@gyne.med.osaka-u.ac.jp (T.K.); 2 Department of Obstetrics and Gynecology, Osaka Rosai Hospital, 1179-3 Nagasone-cho, Sakai 591-8025, Osaka, Japan; ytanaka@gyne.med.osaka-u.ac.jp; 3 Ogata Family Clinic, 2-3 Matsunouchi-cho, Ashiya 659-0094, Hyogo, Japan; minekawa@ogatafamilyclinic.com; 4 Department of Children’s and Women’s Health, Division of Health Sciences, Graduate School of Medicine, Osaka University, 1-7, Yamadaoka, Suita 565-0871, Osaka, Japan; endo@gyne.med.osaka-u.ac.jp; 5 Department of Clinical Psychology, Graduate School of Human Sciences, Osaka University, 1-2, Yamadaoka, Suita 565-0871, Osaka, Japan; khirai@grappo.jp; 6 Center for Public Health Sciences, National Cancer Center, 5-1-1, Tsukiji, Chuo-ku 104-0045, Tokyo, Japan; tomnakay@ncc.go.jp

**Keywords:** cervical cancer, screening rate, HPV vaccine, vaccination status, health consciousness

## Abstract

Women born between 1994 and 1999 achieved high vaccination rates for human papillomavirus (HPV); they are now reaching the age of cervical cancer screening programs in Japan. In this study, we aimed to investigate the health awareness of HPV-vaccinated and unvaccinated women and to create tailored leaflets recommending cervical cancer screening for each. Surveys on the cancer screening rates for HPV-vaccinated and unvaccinated women aged 20 and 21 have demonstrated that the rate was significantly higher (*p* < 0.01) in vaccinated (6.2%) than in unvaccinated women (3.1%). Next, interviews and Internet questionnaires clarified that there was a trend that vaccinated women have a better health consciousness than the unvaccinated ones, and that in unvaccinated women, their willingness to receive cervical cancer screening was significantly enhanced by the fear of developing cancer. Finally, in a prospective study, the increase in the screening rate for both vaccinated and unvaccinated groups after they read tailored leaflets, from 6.4% to 7.4% and from 3.9% to 5.1%, respectively, was not statistically significant compared to the groups provided with a standard reminder letter. Cervical cancer control measures might be enhanced by recommending cervical cancer screening in ways better tailored to HPV vaccination status.

## 1. Introduction

It is now widely accepted that the vast majority of cervical cancers can be easily prevented by timely human papillomavirus (HPV) vaccination and regular cervical cancer screening. As evidence, the incidence and mortality rates of cervical cancer continue to decline in the USA and UK, where cervical cancer screening rates are relatively high [1]. A significant reduction in cervical cancer by human papillomavirus (HPV) vaccination has recently been reported in Finland [2]. In Sweden, for women 10 to 30 years old, quadrivalent HPV vaccination was associated with a substantially reduced risk of invasive cervical cancer [3].

There is a statistical prediction in Australia that if current high-coverage vaccination and screening rates are maintained, cervical cancer can be eliminated by 2028, at an elimination threshold of four new cases per 100,000 women annually [4].

Japan’s Ministry of Health, Labor, and Welfare (MHLW) recommends that all women aged 20 and older should be receiving screening every two years to achieve the earliest possible detection of cervical cancer. Despite this recommendation, the overall screening rate in Japan currently remains at roughly 40%, and it is extremely lower in the youngest generations: 10%, 10–20%, and 10–30%, at age 20–24, 25–29, and 30–39, respectively [5].

Vaccination against HPV-16 and HPV-18 in Japan was first introduced for girls aged 13–16 in fiscal year (FY) 2010. Because HPV-16 and -18 cause about 70% of cervical cancers in the world, bivalent or quadrivalent HPV vaccines which can prevent HPV-16 and -18 infections are expected to reduce cervical cancer incidence by around 70% [6]. A nine-valent HPV vaccine should reduce cervical cancer further, to around 90%, by preventing infections with additional oncogenic strains of HPV-6, -11, -16, -18, -31, -33, -45, -52, and -58 [7].

Women who were born between 1994 and 1999 soon enjoyed rates of vaccination protection approaching 70% [8,9,10]. However, due to media reports in early FY 2013 on alleged adverse vaccination events, the MHLW suspended its recommendation for HPV vaccination. The Ministry’s “no-recommendation” policy has continued now for going on eight years, which has resulted in the collapse of the vaccination rate to near zero [11]. Cumulative vaccination coverage among girls born in FY 2000 was 14.3%; however, it was markedly decreased to 6.1% among girls born in FY 2001, and it is now less than 0.1% [12].

We found, in recent data from Osaka, Japan, that leading up to the year 2000, there had been a considerable decline in age-adjusted incidence of cervical cancer (per 100,000), but after 2000, this trend has been reversed and it has been significantly increasing to date (Annual Percent Change(APC) = 3.8, 95% CI: 2.7 to 4.8) [13]. We find an urgent need to reverse these upward trends in both incidence and mortality as early as possible. Under the current situation of a lack of an aggressive HPV vaccination program in Japan, improvement in the nationwide screening rate of cervical cancer is our only alternative. Teenage females who did or did not benefit from the high HPV vaccination rate circa 2010–2013 are now becoming targets for Japan’s cervical cancer screening program. As unvaccinated women possess a greater risk of future cervical cancer, it is clear that engagement in education and encouragement of an effective lifetime screening program are mandatory.

To date, it has not been studied whether HPV vaccination status has an impact on subsequent cancer screening participation. A previous study implied that the screening rate was relatively higher in HPV-vaccinated women than in unvaccinated ones [14]; however, the unvaccinated group in the study was significantly older than the vaccinated group (*p* < 0.001), and therefore, it seemed that an age distribution difference may have affected the result. Another report was our own interim analysis [15]. For our present study, we restricted the study population to ages 20 and 21 in order to eliminate age distribution bias.

In the present study, we have first compared screening rates between HPV-vaccinated and unvaccinated women of the same age so that age bias can be excluded. We also compared these two groups through interviews designed to assess their knowledge and awareness of cervical cancer, including how to protect against it. We subsequently analyzed the effectiveness of tailored leaflets recommending cervical cancer screening which were more tailored to the individual’s vaccination status.

## 2. Methods

### 2.1. Survey of Cervical Cancer Screening Rates in HPV-Vaccinated and Unvaccinated Women

Data for cervical cancer screening rates in women aged 20 or 21 for FY 2015–2016 were derived from two Japanese cities, Toyonaka and Iwaki. In Japan, cervical cancer screening programs start at age 20 and screening with cytology is provided every two years. Women who have missed attendance during their target year for screening are still eligible for the exam the next year; thus, in the present study, we included the examinees aged 21 who had missed their screening at age 20. When we compared cervical cancer screening rates in vaccinated and unvaccinated women, data on individual vaccination status were confirmed by records in local healthcare departments because in a previous study, 11–20% of women misremembered their vaccination history [16]. We targeted only females who were born in FY 1995–1996, who received HPV vaccination, and who underwent cervical cancer screening at age 20 or 21 in FY 2015–2017 in Toyonaka or Iwaki, since data were not available in the local departments for those who moved to these cities after having received HPV vaccination somewhere else.

### 2.2. Interview Survey of the Vaccinated and the Unvaccinated Women

As a qualitative study, in order to find out the differences between vaccinated and unvaccinated females, 60-minute semi-structured individual interviews were carried out with 10 women aged 20 or 21 on 8, 10, and 15 of February 2018. Interviewees were randomly selected from a commercial survey panel after an initial screening survey; five of the women had an HPV vaccination and five did not. Health consciousness, knowledge about cervical cancer and means of protection against it, and attitude toward cervical cancer screening were surveyed. Based on the interviews above, we created tailored recommendation messages of cervical cancer screening for vaccinated and unvaccinated women. Although the number of the interview survey targets was small, a quantitative assessment was performed with the following Internet survey.

### 2.3. Internet Survey of Vaccinated and Unvaccinated Women Aged 20 or 21

We subsequently performed an Internet survey of 512 females aged 20 or 21, which included 206 vaccinated and 206 unvaccinated women, on 28 and 29 June 2018, as a quantitative study to evaluate recommendation messages developed by assessing whether messages changed women’s willingness to have cervical cancer screening or not. Prior to the survey, all women listed on the Internet survey panel were prescreened with questions about their age and agreement to participate in the larger study. Internet-based questionnaires were sent to women who satisfied our criteria; respondents continued to be accepted until the number of responses from both vaccinated and unvaccinated women reached over 200 per group. Questionnaires covered their health consciousness, knowledge of cervical cancer and how to protect against it, and attitudes toward cervical cancer screening. We also asked whether their willingness to participate in cancer screening was changed by the recommendation messages we provided, depending on their HPV vaccination status. On the basis of this Internet survey, we developed two different information leaflets regarding cervical cancer screening tailored for vaccinated and unvaccinated women, so as to evaluate the effectiveness of our recommendation tools.

### 2.4. A Prospective Comparison of Different Recommendation tools for Screening According to the Respondent’s HPV Vaccination Status

**Figure 1:**
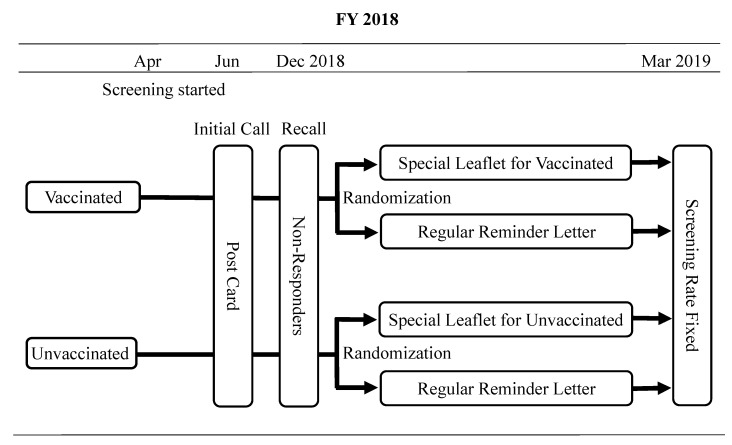
The study schema of randomized comparison of information leaflets tailored to human papillomavirus (HPV) vaccination status versus the standard reminder letter.

Study Targets, Interventions, and Study Design: Using the information leaflets we created as above, our main survey was conducted in FY 2018 in Hirakata City (population 400,000), Osaka, Japan. Every year in June, the municipality of Hirakata usually sends a postcard of invitation with a voucher for free cervical cancer screening to all women reaching age 20 that year; then, in December, the municipality sends reminder letters to anyone who has not yet attended their examination by that time. It was the women who had not attended a cancer screening by 30 November 2018 who were targeted in our main survey, and two groups—vaccinated and unvaccinated—were further divided by the 1:1 principle into two sub-groups on 1 December 2018, resulting in four groups total (Figure 1). Half of the vaccinated group received screening information leaflets specifically tailored to women who had already received a vaccination, and the other half of the group was provided with a municipal reminder letter in the standard format. Similarly, half of the unvaccinated women received a different screening information leaflet which was more encouraging to unvaccinated women; the other half of the group was provided the standard municipal reminder letter. The HPV vaccination status of each attendee was confirmed by local healthcare department records. Note that women who moved to Hirakata after having received HPV vaccination in other cities were excluded from our survey, as their data on vaccination were not available locally.

A screening rate of 6.2% was expected among the vaccinated women, as shown in Table 1, and this rate was expected to increase 1.7-fold, as shown in Table 3B (20.9% to 34.5%). The minimal number of cases needed to asses this increase by a power of 0.8 and α: 0.05 was calculated to be 510 for each arm. The target numbers in the present study, 687 receiving regular reminder letters and 688 receiving special leaflets in the vaccinated group, are thought to be sufficient to analyze.

Outcome: The screening rate achieved after being provided each recommendation tool was the main outcome measure for our study.

### 2.5. Statistical Analysis

Ratio variables were compared by the chi-square test, and a *p*-value of less than 0.05 was defined as being statistically significant.

### 2.6. Informed Consent and Ethical Approval

This study was approved by the Ethics Committee of the Osaka University Hospital (ethical code: 13261-10). All participants provided informed consent by clicking the “I agree” button during the screening step for both the interview survey and the Internet questionnaires. All data provided by local governments were anonymized, with no personal information. This study was performed in accordance with all relevant guidelines and regulations in effect at the time governing procedures conducted in Japan.

## 3. Results

### 3.1. Cervical Cancer Screening Rates in Vaccinated and Unvaccinated Women at Age 20–21 

Among 5587 women born in FY 1995 and FY 1996, and targeted for cervical cancer screening at age 20 and 21 in FY 2015 and FY 2016, in Toyonaka and Iwaki, the overall HPV vaccination rate was 66.2% (3697/5587). The attendance rates for cervical cancer screening in vaccinated and unvaccinated women showed similar trends in the two cities (Table 1). In total, the cervical cancer screening rate in the vaccinated group was twice as high as that of the unvaccinated group, 6.2% versus 3.1%, respectively (230/3697 versus 59/1890; *p* < 0.01) (Section 2.1).

### 3.2. Interview Survey for Preparation of Recommendation Message

The initial interview survey indicated that women in the vaccinated group were more health-conscious: all five thought that cervical cytology is still necessary even after vaccination. They showed their intention to attend a check-up when they received letters from the municipality recommending cervical cancer screening. In contrast, five unvaccinated women showed lower awareness of the necessity for either vaccination or cervical cancer screening, indicating that they were more confident with their health and more skeptical about medical care in general. Unvaccinated women also expressed their fear of injection as a reason to avoid vaccination and fear/dislike of a gynecological examination as a reason for non-attendance of screening. Vaccinated respondents were motivated for screening; however, they admitted that they would have been more willing to attend it if they had been better informed of the screening procedure, i.e., that it was relatively painless and easy to complete. They responded that a leaflet with a better explanation could have guided the women to improve their willingness for undertaking an examination. All these responses led us to create new recommendation messages for vaccinated women, focusing on the details of the screening procedures. In contrast, we found that there was also a necessity for a customized leaflet for highly skeptical unvaccinated women to better inform them of their susceptibility to HPV infections which could lead to a high risk for cervical cancer, the severity of cervical cancer, an almost certain loss of fertility in the case of an advanced cancer, and even loss of their own life (Section 2.2).

### 3.3. Internet Survey for Preparation of Information Tools

The characteristics of the 206 vaccinated and 206 unvaccinated participants in the Internet survey and their answers regarding health consciousness are shown in Table 2. There were no particular differences in marital status, childbearing status or presence of housemates between the two groups, although the employment rate was higher in the unvaccinated group (*p* < 0.001). The percentages of sexual intercourse experiences were not significantly different between the two groups: 55.3% and 59.1% in vaccinated and unvaccinated groups, respectively. The smoking rate was also similar. The percentage of women who had the appropriate knowledge that HPV is the most common cause of cervical cancer was surprisingly low in both groups: 33.5% in the vaccinated and 29.6% in the unvaccinated group. There was a relatively low percentage of women in both groups who knew that HPV is transmitted through sexual intercourse. There was a significant difference between the two groups in their perceptions about cervical cancer, in terms of the fact that it might result in death (66.0% vs. 54.9%, *p* = 0.027). Women in the vaccinated group had a greater intention to receive cervical cancer screening than those in the unvaccinated group (67.0% vs. 53.4%, *p* = 0.0064). Health consciousness and family behavior seemed more noticeable in the vaccinated group than in the unvaccinated, although it showed no statistical significance. Subsequently, two different recommendation messages for cervical cancer screening were presented to the survey participants. In the message for the vaccinated group, there were seven sentences, as follows: 1: The leading cause of death for women aged 20 to 29 is cancer (except for suicide); 2: The number of deaths from cervical cancer is 1.7 times higher than the traffic-related death toll; 3: The cause of cervical cancer is HPV, a common virus that most women get infected by; 4: Females aged 20 or older are recommended to receive cervical cancer screening every two years; 5: Cervical cancer screening can detect premalignant cells; 6: Cervical cancer screening at age 20 is offered free of charge; 7: Because the HPV vaccine is not 100% protective, women who have been vaccinated are still advised to follow regular check-ups. For the unvaccinated women, the message contained three more sentences, Nos. 7–9, in addition to the previous sentences above: 7: Unvaccinated women are not protected from HPV; 8: Cervical cancer patients initially have no apparent symptoms; 9: There has been a case of a 24-year-old woman whose fetus and uterus had to be removed because of her cervical cancer. The same facts about cervical cancer and its prevention, as well as details of the cervical cancer screening procedure, were included for both vaccinated and unvaccinated women (Section 2.3).

Table 3 shows whether or not the intention to receive cervical cancer screening was enhanced by reading our various recommendation messages, and whether or not the women would undergo screening within two years if they changed their mind. Women in the vaccinated group were more motivated for cancer screening after reading the messages than those in the unvaccinated group (58.3% vs. 49.0%, *p* = 0.075). In both vaccinated and unvaccinated women, their intention to receive screening within two years increased dramatically after reading the messages, from 20.9% to 34.5% (*p* = 0.0029) in the vaccinated group and from 18.9% to 32.0% (*p* = 0.0032) in the unvaccinated group.

Participants answered with various reasons regarding how the recommendation messages changed their attitude towards cervical cancer screening, as shown in Table 4. For both vaccinated and unvaccinated groups, the common responses were: “Because I found out that cervical cancer screening is free”, “Because I found out that cervical cancer screening is recommended for ages 20 or older”, and “Because I understood that I am at certain risk of having cancer”. The unvaccinated group were more likely to express their fear of cervical cancer as one of the reasons, and the vaccinated group, in contrast, responded that they recognized the importance of screening even after vaccination.

### 3.4. Information Leaflets for Cervical Cancer Screening Tailored for Vaccination Status

We combined the recommendation messages with the responses from the Internet survey and thus created information leaflets better tailored to meet different vaccination statuses (Supplementary Materials). In the leaflet geared to vaccinated women, the importance of screening even after vaccination was emphasized more, while in the leaflet for unvaccinated women, informing them of the fear of having cancer was the highest priority (Section 2.3).

### 3.5. A Randomized Comparison of Different Recommendation Tools for Screening Tailored for Different HPV Vaccination Status

For Hirakata City, where we conducted our main survey, the cervical cancer screening rates for vaccinated and unvaccinated women at age 20 or 21 are shown in Table 5. By the 1:1 principle, 687 were allocated to the group receiving regular reminder letters and 688 were allocated to the group of vaccinated women receiving special leaflets. In the same random way, 334 were allocated to the group receiving regular reminder letters and 332 were allocated to that receiving special leaflets among the unvaccinated women. Among the vaccinated group, 7.4% of women who read our tailored leaflet underwent cervical cancer screening, compared to 6.4% who received only the ordinary reminder letter from their local government. It was similar in the unvaccinated group, where more women attended the screening after they received our leaflet than the women receiving ordinary reminder letters (5.1% vs. 3.9%). Despite the lack of statistically significant difference between the type of recommendation tools and the screening rates, there was an increase in screening rate in both groups after their reading of the special leaflets that had been adjusted to their vaccination status, when compared to results from the standard reminder letter (Section 2.4).

## 4. Discussion

It is well known that cervical cancer remains one of the most common cancers in women, even though it can be effectively prevented by partaking in early HPV vaccination and screening starting at a younger age. The individualized risk of future cervical cancer is almost completely determined by two factors: personal HPV vaccination status and willingness to undergo screening. It is obvious that women who are neither HPV-vaccinated nor ever screened are at much higher risk than women who are HPV-vaccinated and continuously screened. Considering the fact that in Japan, the age-adjusted incidence of cervical cancer shows a decreasing trend, primary prevention interventions should be undertaken with no further delay.

In Japan, due to the continued suspension of the governmental recommendation of the HPV vaccine, the relative risks of infection with HPV and lifetime incidence of cervical cancer were predicted to differ greatly depending on a woman’s FY of birth [17,18]. To date, it has not been studied whether a woman’s HPV vaccination status has an impact on subsequent cancer screening participation.

In Western countries, including the United States and the United Kingdom, the cervical cancer screening rate is roughly 80%; however, in Japan, it is only 25% [19]. What is particularly problematic is that the screening rate for women aged 20–29 years is less than 10% [20].

This is the first study that has investigated the reasons behind the higher screening rates occurring among vaccinated women in Japan compared to the dismal rate that is occurring in unvaccinated women. Results of our Internet survey suggest that in terms of knowledge, consciousness, and behavior, vaccinated women were more motivated than the unvaccinated women, and, interestingly, families of vaccinated women also showed higher health consciousness. Long-term individual health consciousness is usually developed in relation to surrounding circumstances, such as family influence. It is sometimes difficult to improve the health literacy of “unhealthy” families; however, promoting the wellness of one family member could contribute to the health consciousness of others. Thus, personalized tools to promote health literacy, such as information leaflets tailored to vaccination status, could be incorporated into healthy family lifestyles.

In this study, we have clarified what has been keeping unvaccinated women away from vaccination and screening programs and have identified several pertinent points that should be emphasized in updating our tailored information leaflets in the future. For example, a free or low-cost fee for cervical cancer screening appealed to both vaccinated and unvaccinated women, which encouraged them to undergo examinations. Promoting fear of cancer is another efficient way to lead unvaccinated women to want to receive screening. In contrast, the vaccinated women were more motivated by the knowledge that cervical cancer screening is still required to further reduce their cervical cancer risk, even after HPV vaccination. It is essential that we better inform women, according to their individualized needs, of the importance of screening, and this policy could make screening more systematic and cost-effective. We believe that these approaches will result not only in a higher protection rate from cervical cancer but also in an improvement of overall health consciousness and behavior.

On the other hand, there are some limitations to our study. We obtained a quite disappointing result when we found that the screening rate in vaccinated women was only 6.2%, although that was still significantly higher than for the unvaccinated women. The awareness and motivation of women for screening was increased by our tailored information leaflets; however, the screening rate did not show a statistically significant change and remained under 10%, even after the leaflets.

This frustrating situation might be partly because the leaflets were sent as a reminder to those who already had delays in attendance, suggesting that they remained reluctant to undergo examination compared to women who responded to the initial call. Hence, the result could have been different if the leaflets were sent earlier, instead of the initial call. Further studies are needed to establish more effective recommendation tools offered at the right moment to improve the screening rates. Another limitation of our study was that the research was targeted only at women aged 20 or 21. It is still unclear whether the intention to receive cervical cancer screening could still be affected by their previous history of HPV vaccination when they become older than 20 or 21. It should be investigated whether the information leaflets we created to complement HPV vaccination status could be effective for those already older than 21. Increasing the awareness of cervical cancer of younger women could contribute to improving screening rates and promoting overall life-long health consciousness; hence, future research should be planned for younger teenagers so as to initiate health education at an earlier stage of life.

Our study demonstrated that unvaccinated women significantly tend to not be fully aware of preventive health measures, which subsequently caused their lower cancer screening rate. Further research will be needed to establish an optimal cancer screening program in a way that facilitates women with different health consciousness levels and status to continue their attendance.

In this study, we clarified the differences in health consciousness between HPV-vaccinated and non-vaccinated women through interviews and Internet surveys and showed the possibility of increasing the rate of cervical cancer screening by adopting behavioral science methods based on these findings. In particular, unvaccinated and less health-conscious women are at particularly high risk of cervical cancer because they tend not to undergo cancer screening as well. It is necessary to encourage them to undergo cervical cancer screening. In this study, we were able to clarify these health disparities. In Japan, as in many countries where the HPV vaccine has been introduced, women of the vaccinated generation are becoming eligible for cervical cancer screening, and cervical cancer control measures will be enhanced in a more detailed manner by recommending cervical cancer screening according to their individualized HPV vaccination status. In the next phase of our studies, we would like to make the recommendation of leaflets for cervical cancer screening according to HPV vaccination status more effective to be utilized in regular cervical cancer screening programs.

## 5. Conclusions

In conclusion, the increase in the screening rate for both vaccinated and unvaccinated groups after they read tailored leaflets from 6.4% to 7.4% and from 3.9% to 5.1%, respectively, was not statistically significant compared to the groups provided with a standard reminder letter in a prospective study; thus, cervical cancer control measures might be enhanced by recommending cervical cancer screening in ways better tailored to HPV vaccination status.

## Figures and Tables

**Table 1:** Cervical cancer screening rates in vaccinated and unvaccinated women, aged 20 and 21, born in fiscal year (FY) 1995–1996, studied in Toyonaka and Iwaki, Japan.

City	Vaccinated	Unvaccinated	*p*-Value
Toyonaka	64/2010 (3.2%)	19/1037 (1.8%)	0.034
Iwaki	166/1687 (9.8%)	40/853 (4.7%)	<0.01
Total	230/3697 (6.2%)	59/1890 (3.1%)	<0.01

**Table 2:** Internet survey results on characteristics and the health consciousness and behavior.

Characteristics	Vaccinated	Unvaccinated	*p*-Value
	(*n* = 206)	(*n* = 206)	
Marriage Status			0.72
Married	19 (9.2%)	16 (7.8%)	
Unmarried	187 (90.8%)	190 (92.2%)	
Childbearing			1
Yes	17 (8.3%)	16 (7.8%)	
No	189 (91.7%)	190 (92.2%)	
Housemate			0.18
Yes	143 (69.4%)	156 75.7%)	
No	63 (30.6%)	50 (24.3%)	
Jobs			<0.001
Employed	22 (10.7%)	52 (25.2%)	
Housewives	184 (89.3%)	154 (74.8%)	
(including students)			
Sexual Experience *			0.46
Yes	105 (55.3%)	107 (59.1%)	
No	85 (44.7%)	74 (40.9%)	
Smoking			0.53
Currently smokes	7(3.4%)	12 (5.8%)	
Has smoked	26 (12.6%)	25 (12.1%)	
Never smoked	173 (84.0%)	168 (81.6%)	
Knowledge about cervical cancer			
Cause of cervical cancer is predominantly HPV			0.46
Yes	69 (33.5%)	61 (29.6%)	
No	137 (66.5%)	145 (70.4%)	
HPV is transmitted by sexual intercourse			1
Yes	60 (29.1%)	59 (28.6%)	
No	146 (70.9%)	147 (71.4%)	
Cervical cancer has been increasing in younger generations			0.072
Yes	130 (63.1%)	111 (53.9%)	
No	76 (36.9%)	95 (46.1%)	
Cervical cancer treatment possibly affects fertility			0.16
Yes	164 (79.6%)	151 (73.3%)	
No	42 (20.4%)	55 (26.7%)	
Cervical cancer may result in death			0.027
Yes	136 (66.0%)	113 (54.9%)	
No	70 (34.0%)	93 (45.1%)	
Health consciousness and behavior			
Intension to receive cervical cancer screening			0.0064
	138 (67.0%)	110 (53.4%)	
Gynecology consultation history *			0.27
Yes	91 (45.3%)	79 (39.7%)	
No	110 (54.7%)	120 (60.3%)	
Have talked about cervical cancer with a family member			0.33
Yes	87 (42.2%)	74 (35.9%)	
No	98 (47.6%)	104 (50.5%)	
Not remember	21 (10.2%)	28 (13.6%)	
Recommended cervical cancer screening by a family member			0.3
Yes	51 (24.8%)	48 (23.3%)	
No	142 (68.9%)	136 (66.0%)	
Don’t remember	13 (6.3%)	22 (10.7%)	
Family members receive cancer screenings			0.16
Regularly	27 (13.1%)	16 (7.8%)	
Irregularly	34 (16.5%)	42 (20.4%)	
Never/don’t know	145 (70.4%)	148 (71.8%)	

*: no—answer was excluded from analysis.

**Table 3:** Effects of the tailored or standard recommendation messages for cervical cancer screening for the vaccinated and unvaccinated women.

(**A**) Change of intention for receiving cervical cancer screening after reading the message			
	Change of intention for receiving screening		
	Enhanced	Unchanged or decreased	*p*-value
Vaccinated (*n* = 206)	120 (58.3%)	86 (41.7%)	0.075
Unvaccinated (*n* = 206)	101 (49.0%)	105 (51.0%)	
(**B**) Intention to receive cervical cancer screening within two years—before and after reading the message			
	Intention to receive cervical cancer screening within two years		
	Before	After	*p*-value
Vaccinated (*n* = 206)	43 (20.9%)	71 (34.5%)	0.0029
Unvaccinated (*n* = 206)	39 (18.9%)	66 (32.0%)	0.0032

**Table 4:** Reasons given for enhanced intention to receive cervical cancer screening.

Vaccinated	Unvaccinated
1. I found out that cervical cancer screening is free.	1. I found out that cervical cancer screening is free.
110/120 (91.7%)	88/101 (87.1%)
2. I found out that I could have cervical cancer myself.	2. I found out how scary cervical cancer is.
108/120 (90.0%)	87/101 (86.1%)
3. I found out that cervical cancer screening is recommended for ages 20 and up.	3. I found out that cervical cancer screening is recommended for ages 20 and up.
105/120 (87.5%)	85/101 (84.2%)
4. I found out that it’s better to receive cervical cancer screening, even though I’ve been vaccinated.	4. I found out that I could have cervical cancer myself.
105/120 (87.5%)	81/101 (80.2%)
5. I found out that more people die of cervical cancer than I thought.	5. I found out what kind of examination the cervical cancer screening is.
103/120 (85.8%)	78/101 (77.2%)
6. I found out how scary cervical cancer is.	6. I found out that more people die of cervical cancer than I thought.
102/120 (85.0%)	77/101 (76.2%)

**Table 5:** Cervical cancer screening rates for women aged 20 or 21 in Hirakata City, in vaccinated and unvaccinated women, after receiving regular reminder letters or specially tailored leaflets according to vaccination status.

City	Regular Reminder Letter	Special Leaflet	*p*-Value
Vaccinated	44/687 (6.4%)	51/688 (7.4%)	0.52
Unvaccinated	13/334 (3.9%)	17/332 (5.1%)	0.46
Total	57/1021 (5.6%)	68/1020 (6.7%)	0.31

## Data Availability

The data that support the findings of this study are available from the corresponding author, upon reasonable request.

## Supplementary Materials

The following are available online at https://www.mdpi.com/2076-393X/9/3/280/s1, Figure S1: Special leaflet for vaccinated women; Figure S2: Special leaflet for unvaccinated women.
