# Supplementary material for: Disparity of Cervical Cancer Risk in Young Japanese Women: Bipolarized Status of HPV Vaccination and Cancer Screening
Source: Vaccines (Basel). 2021 Mar 19;9(3):280. doi: 10.3390/vaccines9030280 (PMC8003385; doi:10.3390/vaccines9030280)
Supplement: Supplementary file 1 [file vaccines-09-00280-s001.zip › Suppl. 1 (Vaccinated Leaflet) translate in English.pptx]

## Slide 1
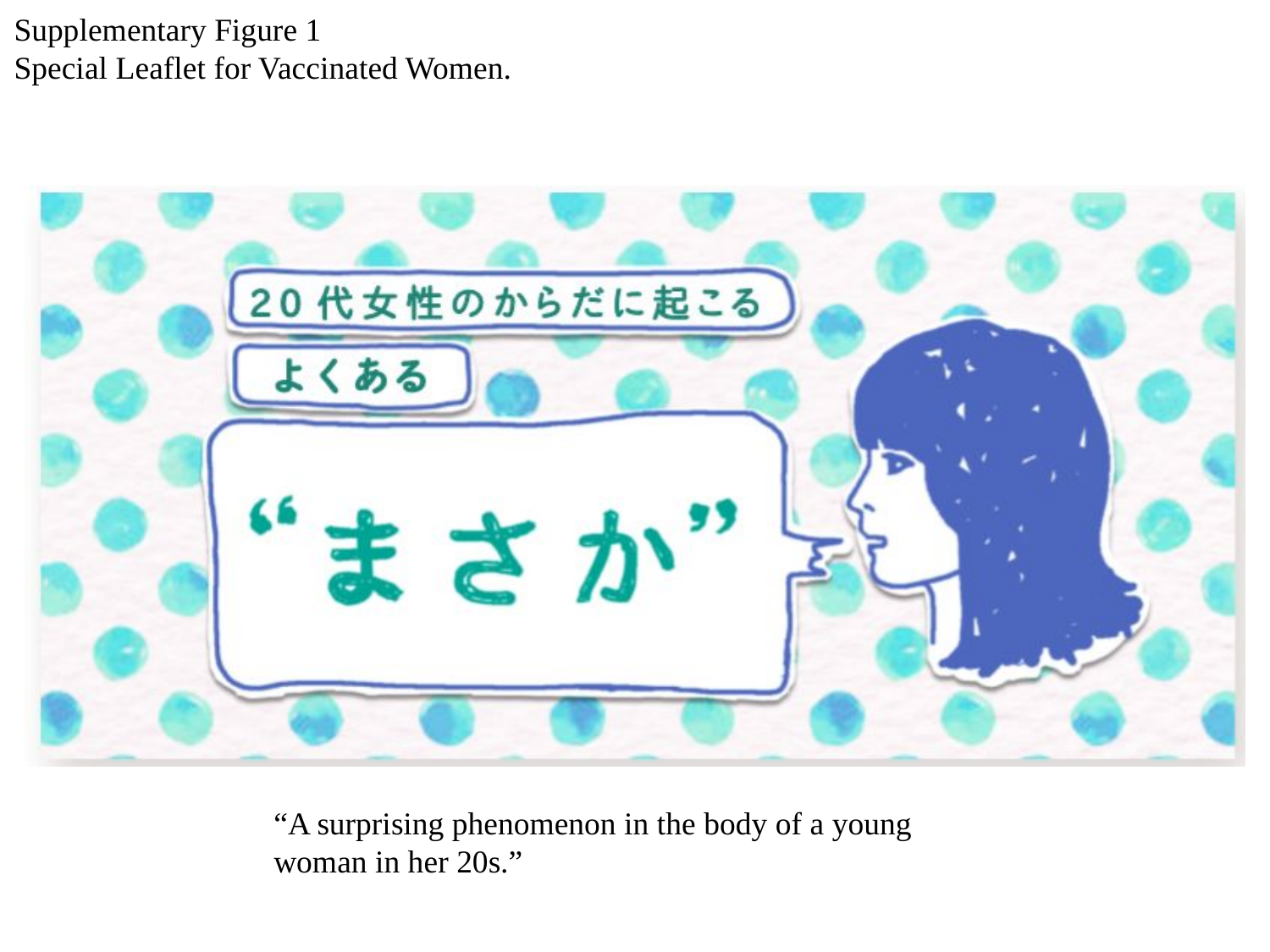

Supplementary Figure 1
Special Leaflet for Vaccinated Women.
“A surprising phenomenon in the body of a young woman in her 20s.”

## Slide 2
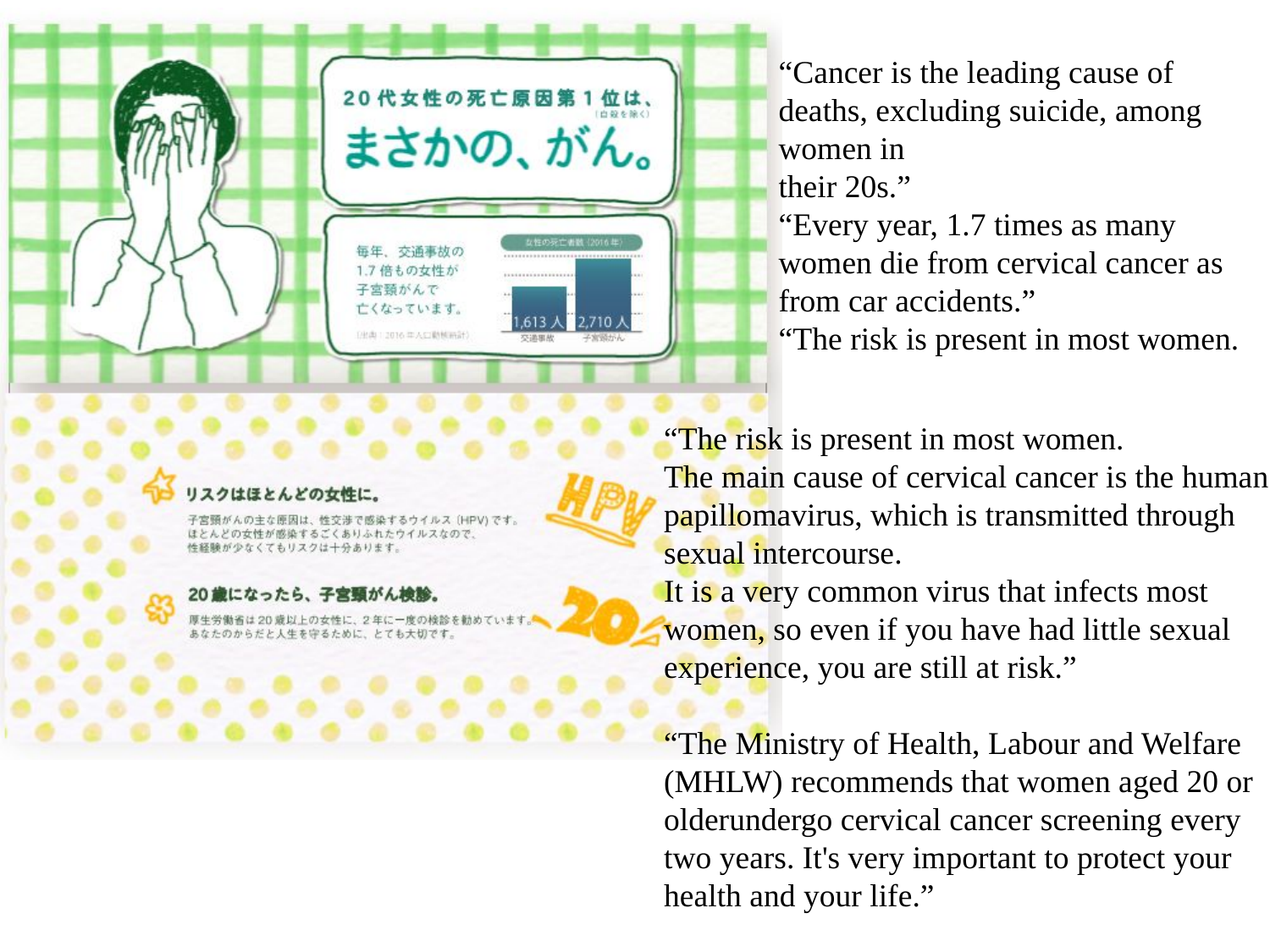

“Cancer is the leading cause of deaths, excluding suicide, among women in 
their 20s.”
“Every year, 1.7 times as many women die from cervical cancer as from car accidents.”
“The risk is present in most women.
“The risk is present in most women.
The main cause of cervical cancer is the human papillomavirus, which is transmitted through sexual intercourse.
It is a very common virus that infects most women, so even if you have had little sexual experience, you are still at risk.”
“The Ministry of Health, Labour and Welfare (MHLW) recommends that women aged 20 or olderundergo cervical cancer screening every two years. It's very important to protect your health and your life.”

## Slide 3
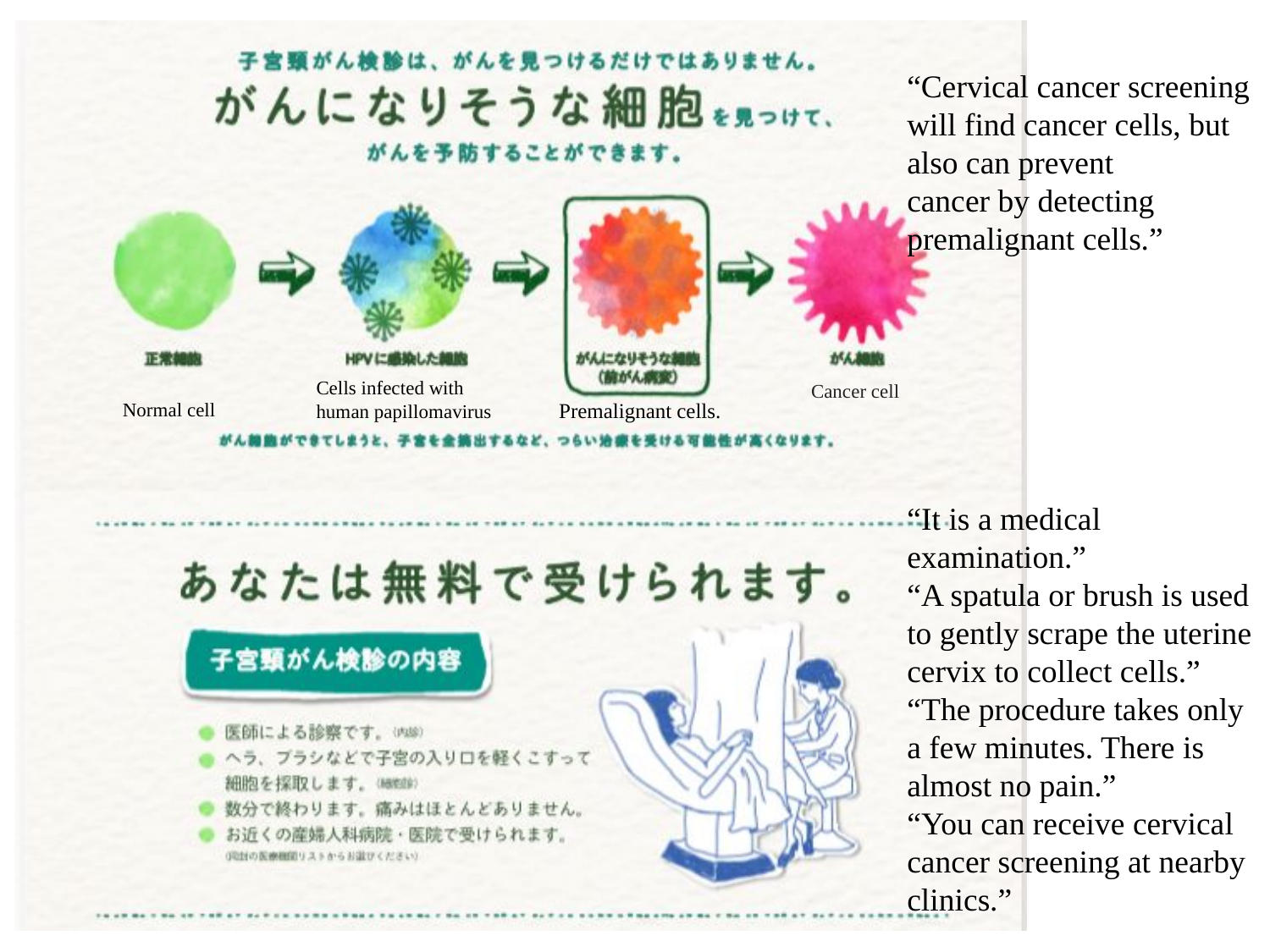

“Cervical cancer screening will find cancer cells, but also can prevent 
cancer by detecting premalignant cells.”
Cells infected with human papillomavirus
Cancer cell
Normal cell
Premalignant cells.
“It is a medical examination.”
“A spatula or brush is used to gently scrape the uterine cervix to collect cells.”
“The procedure takes only a few minutes. There is almost no pain.”
“You can receive cervical cancer screening at nearby clinics.”

## Slide 4
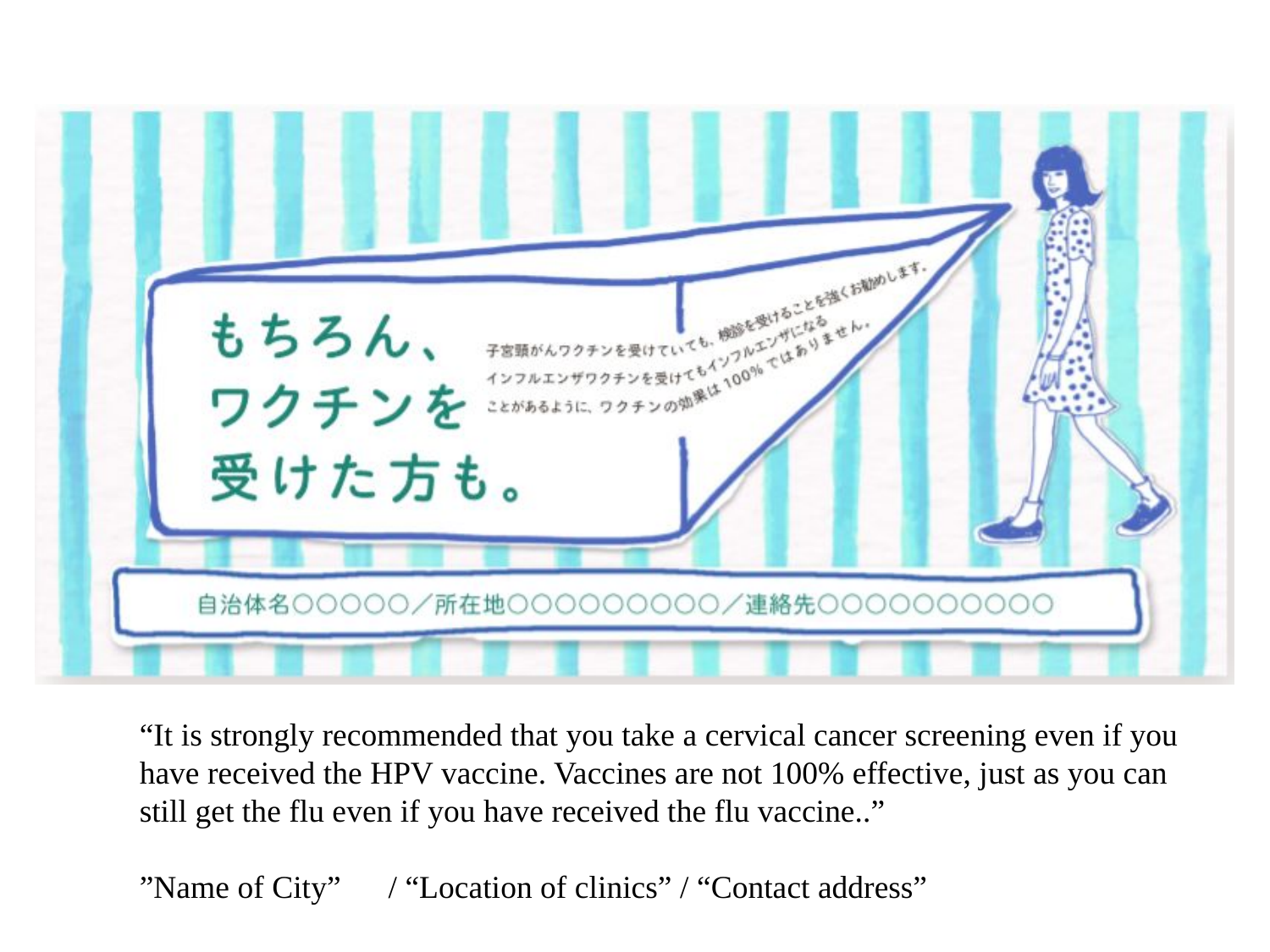

“It is strongly recommended that you take a cervical cancer screening even if you have received the HPV vaccine. Vaccines are not 100% effective, just as you can still get the flu even if you have received the flu vaccine..”
”Name of City”　/ “Location of clinics” / “Contact address”
