# Supplementary material for: Disparity of Cervical Cancer Risk in Young Japanese Women: Bipolarized Status of HPV Vaccination and Cancer Screening
Source: Vaccines (Basel). 2021 Mar 19;9(3):280. doi: 10.3390/vaccines9030280 (PMC8003385; doi:10.3390/vaccines9030280)
Supplement: Supplementary file 1 [file vaccines-09-00280-s001.zip › Suppl. 2 (Unvaccinated) translate in English.pptx]

## Slide 1
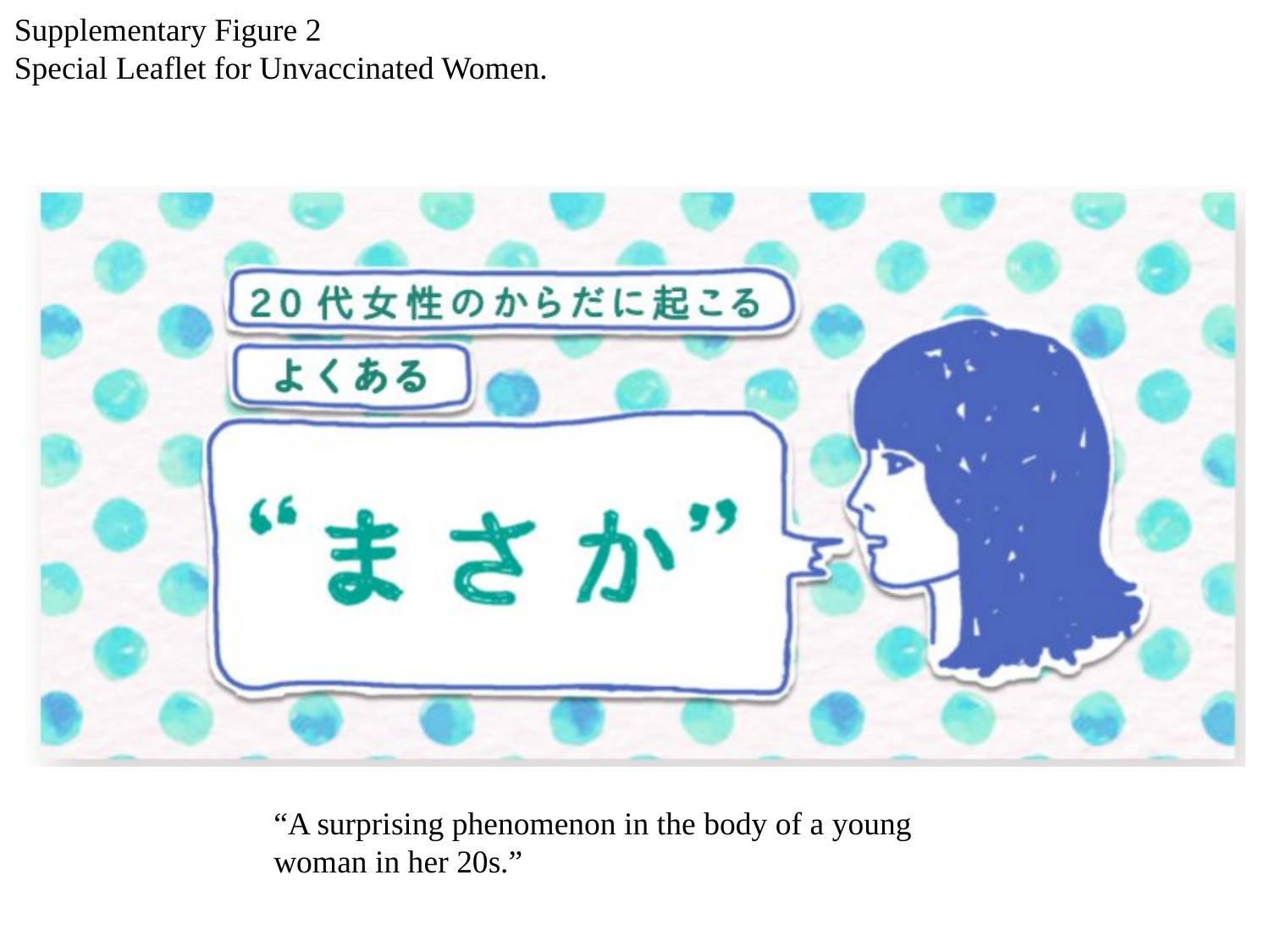

Supplementary Figure 2
Special Leaflet for Unvaccinated Women.
“A surprising phenomenon in the body of a young woman in her 20s.”

## Slide 2
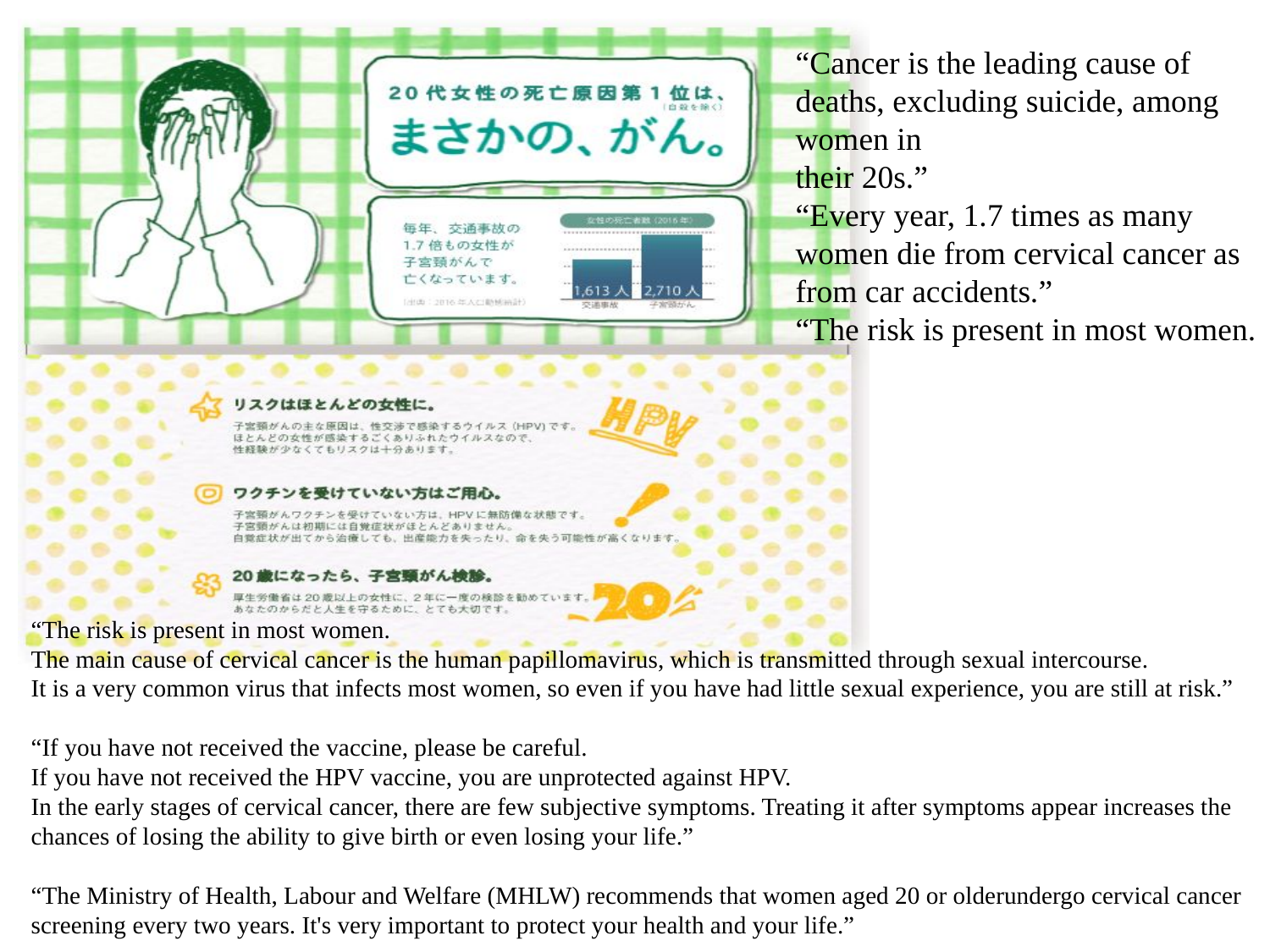

“Cancer is the leading cause of deaths, excluding suicide, among women in 
their 20s.”
“Every year, 1.7 times as many women die from cervical cancer as from car accidents.”
“The risk is present in most women.
“The risk is present in most women.
The main cause of cervical cancer is the human papillomavirus, which is transmitted through sexual intercourse.
It is a very common virus that infects most women, so even if you have had little sexual experience, you are still at risk.”
“If you have not received the vaccine, please be careful.
If you have not received the HPV vaccine, you are unprotected against HPV.
In the early stages of cervical cancer, there are few subjective symptoms. Treating it after symptoms appear increases the chances of losing the ability to give birth or even losing your life.”
“The Ministry of Health, Labour and Welfare (MHLW) recommends that women aged 20 or olderundergo cervical cancer screening every two years. It's very important to protect your health and your life.”

## Slide 3
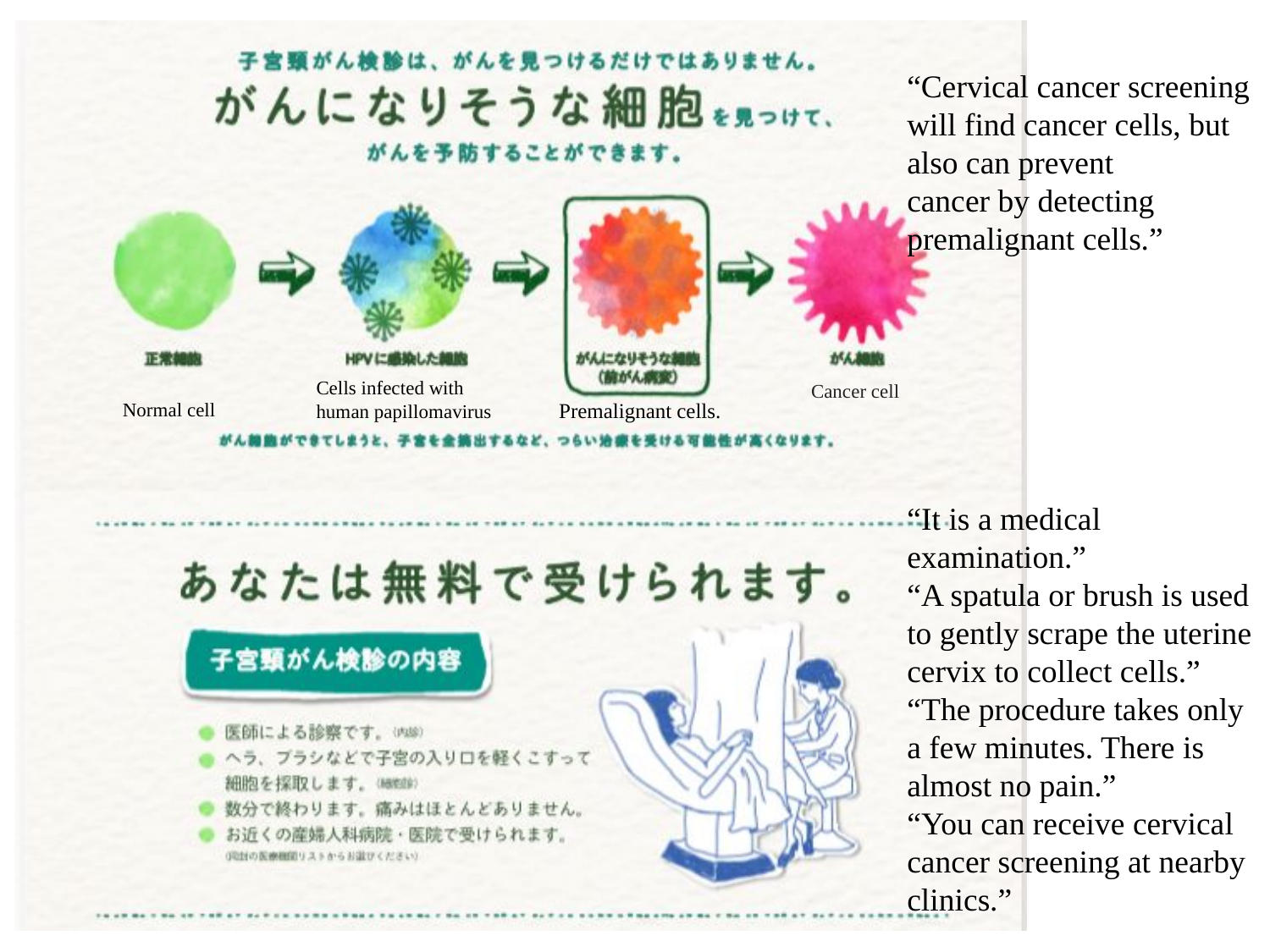

“Cervical cancer screening will find cancer cells, but also can prevent 
cancer by detecting premalignant cells.”
Cells infected with human papillomavirus
Cancer cell
Normal cell
Premalignant cells.
“It is a medical examination.”
“A spatula or brush is used to gently scrape the uterine cervix to collect cells.”
“The procedure takes only a few minutes. There is almost no pain.”
“You can receive cervical cancer screening at nearby clinics.”

## Slide 4
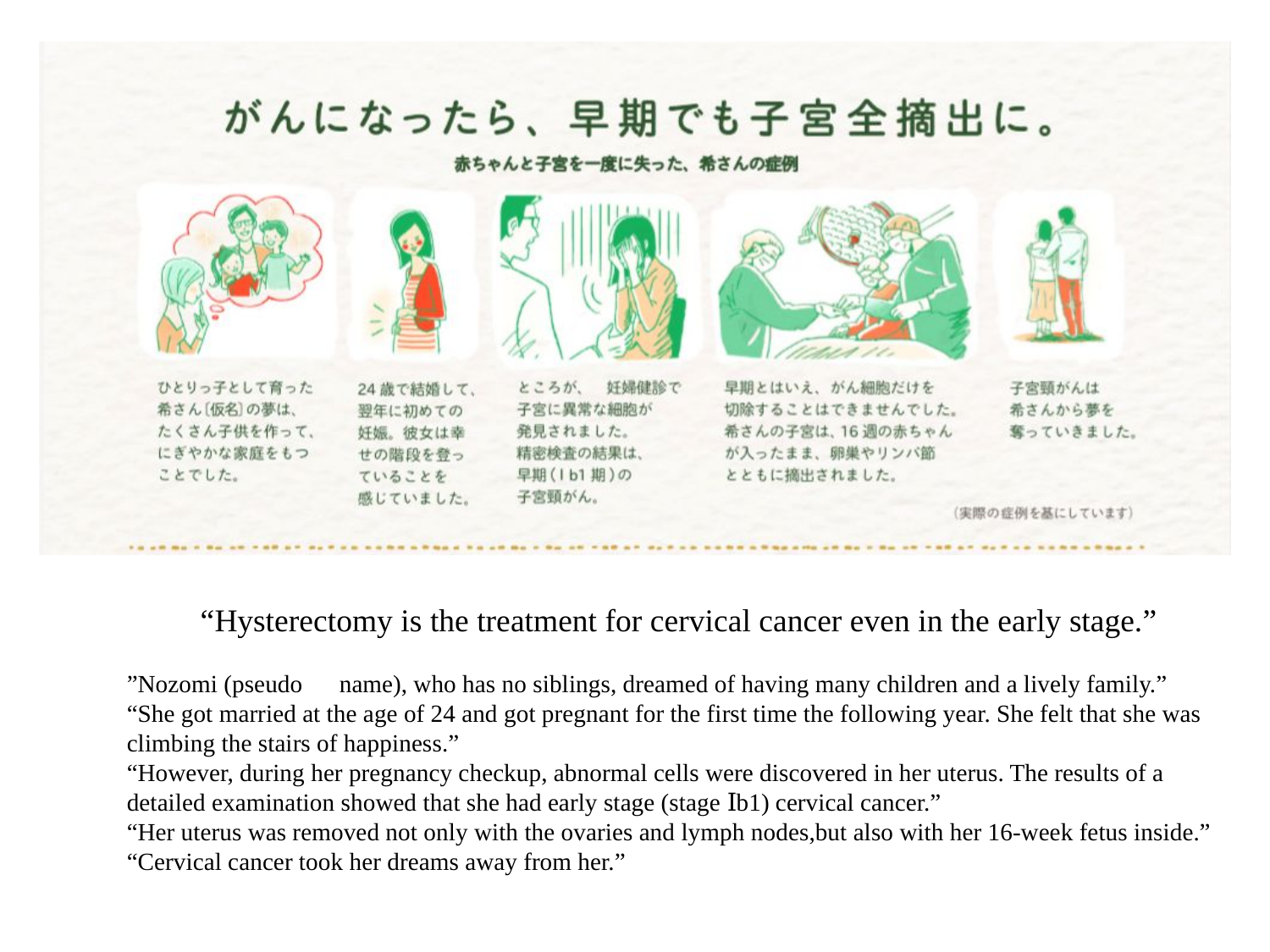

“Hysterectomy is the treatment for cervical cancer even in the early stage.”
”Nozomi (pseudo　name), who has no siblings, dreamed of having many children and a lively family.”
“She got married at the age of 24 and got pregnant for the first time the following year. She felt that she was climbing the stairs of happiness.”
“However, during her pregnancy checkup, abnormal cells were discovered in her uterus. The results of a detailed examination showed that she had early stage (stage Ⅰb1) cervical cancer.”
“Her uterus was removed not only with the ovaries and lymph nodes,but also with her 16-week fetus inside.”
“Cervical cancer took her dreams away from her.”
